# From Triads to Tools: A Comprehensive Review of the Expanding Roles of G-Triplex Structures

**DOI:** 10.3390/molecules30214303

**Published:** 2025-11-05

**Authors:** Mitchell W. Myhre, Malay Kumar Das, Elizabeth P. Williams, Wendi M. David, Sean M. Kerwin

**Affiliations:** 1 Department of Chemistry & Biochemistry, Texas State University, San Marcos, TX 78666, USA; mwm95@txstate.edu (M.W.M.); epw14@txstate.edu (E.P.W.); wdavid@txstate.edu (W.M.D.); 2 Materials Science, Engineering, and Commercialization Program, Texas State University, San Marcos, TX 78666, USA; isq12@txstate.edu

**Keywords:** G-triplex, G-quadruplex, DNAzyme, biosensors

## Abstract

Interest in non-canonical DNA structures continues to grow, in part fueled by the recent discovery of a new structure, G-triplex DNA. Originally proposed as folding intermediates for G-quadruplex DNA, G-triplex DNA has more recently been shown to form from truncated G-quadruplex sequence oligonucleotides and other, specifically designed sequences. In this review, we provide the first, comprehensive survey of G-triplex DNA and RNA, covering the literature up to 2024. We include reports of G-triplex DNA from bulk solution and single-molecule approaches, the structural characterization of G-triplex DNA, and the breadth of oligonucleotide sequences that have been reported to form these structures. The formation of G-triplex RNA structures is also reviewed. The evolving understanding of sequence and environmental effects on G-triplex formation are presented together with challenges due to structural polymorphism and competing formation of multimeric G-quadruplex structures. Hints of the biological relevance of G-triplexes are provided by reports of protein recognition of these structures and their effects on DNA replication in vitro. Interaction of G-triplex DNA with a variety of ligands has been reported, although the search for selective ligands that can distinguish G-triplex from G-quadruplex is on-going. The vast majority of publications in the area have focused on the utilization of G-triplex in biosensing applications, which has shown some advantages compared to G-quadruplex-based systems. These results highlight the potential utility of G-triplex structures in a variety of domains and show its promise in applications in biotechnology, medicine, and research.

## 1. Introduction

**Figure 1:**
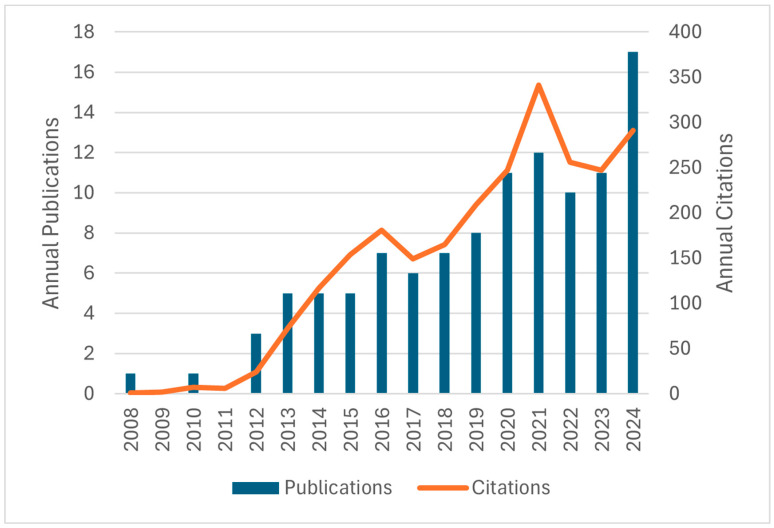
Growth of G-triplex literature over time. Assembled using Web of Science, using final publication years for all articles.

Although DNA is most often considered as a duplex that adopts an anti-parallel right-handed helical structure, there is growing interest in the less common, non-canonical structures that DNA can adopt. These non-canonical DNA structures include left-handed Z-DNA double helixes, i-motifs, three-stranded H-DNA helixes, four-stranded G-quadruplex structures, and cruciform structures [1,2]. Interest in non-canonical DNA structures stems from evidence that these structures play biological roles and may serve as drug targets for drug design [3,4], but also from insights into the roles these structures can play in the design of DNA-based technologies for computation and reconfigurable devices [5]. Considering this interest in non-canonical DNA structures, the discovery of a new non-canonical DNA motif understandably generates much enthusiasm. This is the case for the recently introduced G-triplex (G3) DNA structural motif, which is the subject of this review. Although more than a decade has passed since the initial descriptions of G3 DNA, there has not been a review of the area to map its origins, evolution, and directions. In light of the growing research focused on G-triplex structures, both DNA- and RNA-based (Figure 1), we have set out to provide an initial, comprehensive review of this burgeoning field, attempting to cover the available literature up to 2024, with apologies to any authors who have published in this area that we may have inadvertently missed. We intend for this review to offer a convenient entry to this area for those who are not yet familiar with it as well as a complete report on the origins and evolution of the field for those who are already exploring these structures. We also provide some thoughts on the challenges facing the field and possible future directions.

## 2. Structural Basis of G-Triplex DNA and RNA

There is a close association between the study of G3 DNA and that of G-quadruplex (G4) DNA, particularly in that G3 DNA was first proposed as a folding intermediate for G4 DNA. Therefore, a brief overview of G4 DNA is provided, along with a presentation of the literature concerning the role of G3 intermediates in the folding of G4 DNA and RNA. This section also provides an overview of the still rather limited experimental work on the characterization of G3 structures, with currently only two such structures reported. We provide a discussion of the contexts that favor G3 formation and provide the DNA and RNA sequences that have been reported to form G3 structures. The environmental conditions found to favor G3 formation are presented with an emphasis on cation effects along with a discussion of the stability of these structures compared to analogous G4 structures.

### 2.1. Overview of G-Quadruplex DNA

**Figure 2:**
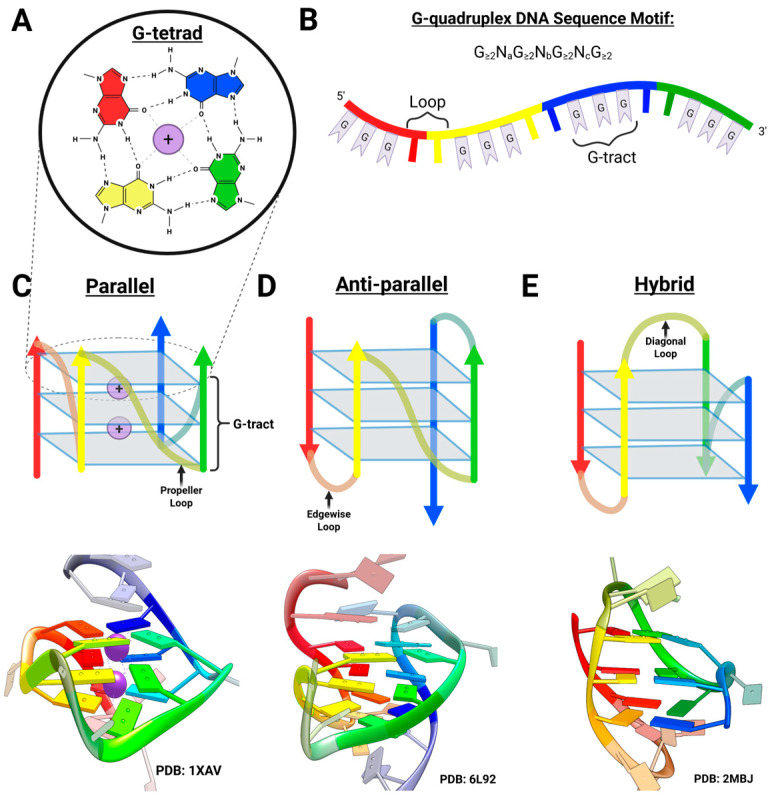
G-quadruplex structure. (**A**) The fundamental structural unit of the G-quadruplex, the G-tetrad. (**B**) The G-quadruplex sequence motif. with each separate run of Gs color-coded from 5′ (red) to 3′ (blue). (**C**–**E**) Cartoon (**top**) and experimentally determined structures (**bottom**) of examples of (**C**) a parallel G-quadruplex, in which all four strands run in the same direction. Dotted circle and lines highlight the G-tetrad portion in the cartoon representations (**D**) An anti-parallel G-quadruplex, in which there are two pairs of antiparallel stands. (**E**) A hybrid G-quadruplex, in which one strand runs in the opposite direction of the other three. Figure created using Biorender.

The self-assembly of guanylic acid into gel-like substances first noted within a decade of the discovery of the double-helical structure of DNA is due to the association of guanine bases to form G-tetrads [6]. G-tetrads are composed of four adjacent coplanar guanines held together by eight hydrogen bonds (Figure 2A). By the 1990s, the identification of the uniform G-rich composition of telomeres hinted at the importance of the G-tetrad [7]. In the following years, structural studies of telomeric DNA established that the G-tetrad serves as the fundamental unit of a class of four-stranded DNA structures known as G4 DNA [8].

In general, formation of an intramolecular G4 DNA requires a sequence fitting the motif G_≥2_N_a_G_≥2_N_b_G_≥2_N_c_G_≥2_ (Figure 2B), where four contiguous “tracts” of two or more guanine residues are separated by “loops” of various lengths. This may be further stabilized in the presence of a metal cation, particularly K^+^, Ca^2+^, Na^+^, and Mg^2+^ [9]. Formation of multimeric, intermolecularly bonded structures is also possible with guanine-rich oligonucleotides and is favored with sequences containing terminal guanines, as well as solutions containing higher DNA and ion concentrations [10].

Early work on human telomeres revealed that ligand-mediated stabilization of telomeric G4 DNAs may inhibit the activity of telomerase, an enzyme responsible for telomere extension that is overexpressed in most cancer cells and contributes to cell immortality [11]. Subsequent studies in the early 2000s revealed a functional G4 DNA within the promoter region of the c-MYC oncogene that affected c-MYC transcription in cancer cells [12,13]. By the end of the decade, the prevalence and non-uniform distribution of putative G4 DNA-forming sequences in the human genome revealed their potential as global gene regulatory motifs [9,14,15]. This expansion of the biological relevance of G4 DNA complicated the aim of targeting these structures with chemotherapeutics. In addition to being highly selective for G4 DNAs in general, optimal therapeutics must be capable of sorting through the nuanced mosaic of the G4 genome to find their specific target with minimal cross-reactivity [16,17].

**Figure 3:**
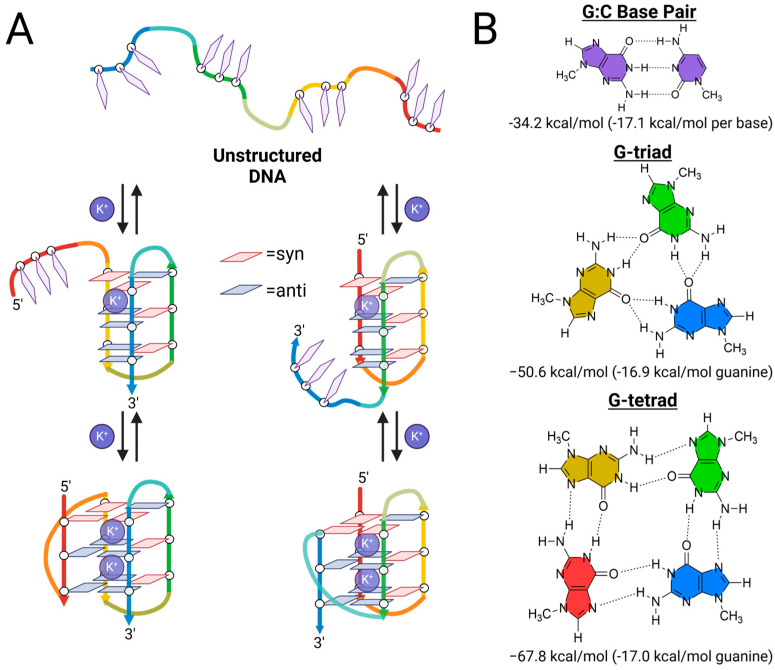
The G-triplex DNA as a folding intermediate of hybrid telomeric G4s. (**A**) Simplified schematic representations of proposed folding pathways of type-1 and type-2 G-quadruplexes from random coil DNA with each separate run of Gs color-coded from 5′ (red) to 3′ (blue). (**B**) Two-dimensional depictions and ab initio stabilities of the G:C base pair, G-triad, and G-tetrad [21]. Figure created using Biorender.

To this end, emphasis was placed on the topological diversity of G4s, which is more reminiscent of protein folding than of DNA duplex formation. Depending on the sequence and cation, a G-quadruplex may form one of several topologies with different strand orientations (Figure 2C–E). As recognition of the functional importance of the DNA folding landscape grew, investigations into the process of G4 folding from single-stranded DNA became imperative to understanding the factors that dictate G4 formation and stability. Through these studies, it was found that the folding process of human telomeric G4 proceeds through at least one intermediate [18,19]. Later investigations into this intermediate’s structural nature established the existence of a new class of guanine-based DNA conformations, the three-stranded G-triplex DNA (Figure 3A).

### 2.2. G-Triplex DNA as a Telomeric Folding Intermediate

Following the suggestion of intermediate states forming within the human telomeric G4 DNA folding process, the earliest proposal of a three-stranded intermediate came from the work of Sugiyama and co-workers [20]. Based on single-molecule Förster resonance energy transfer (smFRET) measurements on 8-bromoguanine substituted variants of the human telomeric G4 sequence, they proposed that the predominate conformations found in K^+^ solution folded through a triple-stranded core structure with three G-triads [20]. To further explore this proposed folding pathway, they subsequently carried out ab initio, molecular dynamics (MD), and fragment molecular orbital (FMO) calculations on the folding pathways of the G4 formed by the human telomeric DNA sequence, Tel22 (Table 1) [21]. Ab initio calculations revealed that the stabilization energy on a per-guanine basis (−16.9 kcal/mol) of the G-triad is very similar to that of a G-tetrad (−17.0 kcal/mol) (Figure 3B). They proposed that stable G-triplex intermediates would contain a preponderance of anti-conformations of the participating guanines, and using MD simulations of the K^+^ complexes they describe two stable G-triplex intermediate structures, each stabilized by a single K^+^ bound proximal to the 5′ G-triad (Figure 3A). They followed up by providing the first direct experimental evidence of a mechanically stable intramolecular G-triplex structure, using circular dichroism (CD), melting temperature analysis, and single-molecule optical tweezers to study a truncated telomeric sequence containing only three G-tracts, Tel21 (Table 1) [22].

Shortly afterward, several other studies corroborated these findings. Solution-based experimental studies of the folding of Tel22 carried out by Lah, Vesnaver and co-workers employing isothermal titration calorimetry (ITC), differential scanning calorimetry (DSC) and CD spectroscopy methods confirmed the formation of a folding intermediate which, based on changes in K^+^ binding, was assigned a G-triplex structure [23]. Contemporary studies by Chaires and co-workers employing CD and fluorescence studies of the thermal melting of Tel22 and related sequences also identified the observed folding intermediate as a G-triplex structure [24]. G-triplex formation, in both four-G-tract- and three-G-tract-containing telomeric sequences, was also confirmed in solution using an α-hemolysin protein ion channel [25]. T-jump relaxation experiments conducted with Tel22 indicated that it folds through intermediates in both sodium and potassium containing buffers, in a DNA concentration-independent manner [26].

**Figure 4:**
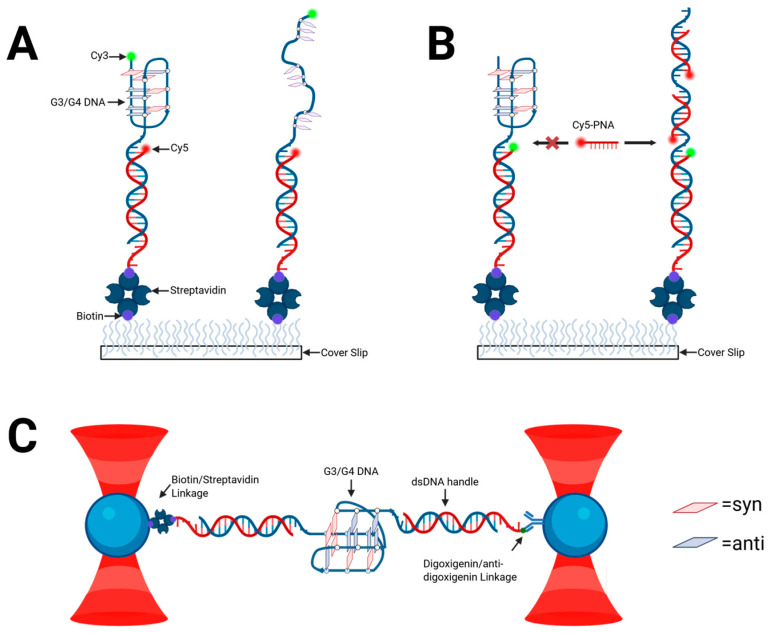
Single-molecule techniques used for the study of telomeric G3 sequences with immobilized DNA strands in red. (**A**) smFRET. (**B**) FRET-PAINT. (**C**) Molecular tweezers. Figure created using Biorender.

As exemplified by CD thermal analysis, DSC, and native polyacrylamide gel electrophoresis (PAGE) of Tel15 in 100 mM K^+^ solution, certain putative telomeric G3 sequences have a tendency towards polymorphism and formation of higher-order aggregates [27]. This presents a challenge to studying telomeric G3s using solution-based methods. Single-molecule approaches such as smFRET, FRET-point accumulation in nanoscale topography (FRET-PAINT), and molecular tweezers (Figure 4) are particularly well-suited to unambiguous characterization of intramolecularly folded G3 DNA. Sugiyama et al. [28] confirmed the existence of G3 as human telomeric G4 DNA folding intermediates in more complex telomeric sequences containing 5–7 GGG repeats, using optical tweezers in potassium-containing buffers [28]. A later experiment by Li et al. using molecular tweezers verified that the human telomeric G3 served as an on-pathway folding intermediate based on extension lengths of the unfolding of telomeric G4 and thermodynamic data consistent with the aforementioned ensemble work [29]. Jiang et al. employed optical tweezers to measure the mechanical stability of G-triplexes formed by Tel15 under various ionic and crowding conditions using PEG 200 [30]. These experiments supported their solution-based findings that G-triplex structures with three G-triads might be stable at physiological temperatures and are particularly stabilized by calcium ions (followed closely by potassium) and molecular crowding agents. The measured unfolding forces (~31–35 pN) and contour length changes matched those previously attributed to G-triplexes, confirming their identity and stability at the single-molecule level [30]. However, subsequent studies by Mitra and co-workers did not find evidence for G3 formation from a truncated human telomeric 3-repeat construct using fluorescence–force spectroscopy [31]. Thus, while there is much evidence supporting the role of G3 structures in the folding of G4, G3 formation likely depends upon a multitude of experimental factors and specific constructs.

Xu-Guang Xi and colleagues utilized smFRET to monitor the folding process of the telomeric G4 sequence and observed four discreet FRET states in sodium-containing buffer, indicating the existence of distinct sodium-stabilized G-triplex and G-hairpin intermediates during G-quadruplex formation [32]. They next studied telomeric G4 constructs with GGG → TTT mutations on either the 5′ or 3′ end, and found that a G-triplex may form on either end of the G4 sequence in the presence of sodium. Building on this, they next applied smFRET to Tel15-based constructs with redesigned fluorophore placements to distinguish between parallel and antiparallel G-triplex topologies, and uncovered evidence for both conformations in the presence of potassium [33]. Based primarily on CD spectroscopy, they further demonstrated that proximal DNA sequences, such as flanking TTA repeats or duplex stems, can reduce G3 DNA folding speed and preferentially stabilize one topology over another. smFRET has also been used to investigate the telomeric sequences of other species. In their smFRET investigation into the structural dynamics of plant telomeres, Wu and coworkers identified a potassium-stabilized intermediate attributed to G3 DNA based on experiments using a mutated sequence containing only three G-tracts [34]. Most recently, FRET-PAINT experiments found that adjacent G4 formation can influence telomeric G3 formation. Based on the binding of a FRET inducing complementary probe, Kodikara et al. found that telomeric G3 formation is most diminished with G4 formation on both its 5′ and 3′ end, to a lesser extent when there is a G4 formed on its 3′ end and unstructured DNA is on its 5′, and is enhanced (relative to 5′ ssDNA) with G4 formation solely on its 5′ end [35]. These findings suggest that G3 formation may be selectively stabilized in certain telomeric contexts, potentially serving as structural regulators or protective elements when G4 formation is limited. Furthermore, they demonstrated that increasing loop length (to TTTTTTTTTTTTA in place of TTA) greatly reduces G3 formation on a single-molecule level.

Solution ensemble-based methods also contributed to the evolving understanding of G3 DNA. CD temperature-jump (T-jump) relaxation experiments conducted with Tel22 in both sodium- and potassium-containing buffers revealed two processes in each case with microsecond, first-order rate constants. These were interpreted as transitions involving a G-quadruplex/G-triplex equilibrium involving two distinct G-quadruplex forms [26]. These authors ruled out other potential folding intermediates or processes primarily based on the concentration and cation nature independence of the observed rates. They further surmise that the G-triplex species are involved in the much slower (time- scale of seconds) interconversion of the two distinct G-quadruplex forms. Based on microscale thermophoresis (MST)-derived dissociation constants of telomeric sequences with mutation in various G-tracts, Zhang et al. proposed the G-triplex composed of G-tracts 1, 2, and 4 as the most energetically favorable G4 folding intermediate in K^+^ buffer [36].

Similar conclusions related to the intermediacy of G-triplex structure in the folding pathway for human telomeric G4 come from molecular dynamics (MD) simulations and various meta-dynamic studies. The folding and unfolding processes for G4 DNA are complicated and likely best represented by kinetic partitioning that involves diverse populations of structures that include G-hairpins, incompletely or mis-folded G-quadruplexes, and cross-like structures in addition to G-triplexes [37,38]. Šponer and co-workers demonstrated that specific topologies of G-triplexes were stable on the µs timescale in MD simulations [39]. They note that parallel-stranded G-triplexes with propeller or dog-ear loops are not stable. In contrast, a number of antiparallel folds are stable; however, those with propeller loops may be less so. These simulations also shed light on the malleability of the G-triad geometry as well as the stabilizing effect of cation binding to G-triplex narrow grooves. However, the computational evidence for G-triplex folding intermediates is complicated by data that either fails to identify such intermediates or fails to support lifetimes for such structures that are compatible with their role as intermediates [40,41,42,43]. This is particularly true for G3 DNA containing only two G-triads, such as those formed from the thrombin binding aptamer (TBA, Table 1). It appears that this discrepancy may be at least partly due to the nature of the molecular mechanics force field employed; Šponer and co-workers note that the stability and MD lifetimes of G-triplex structures derived from the TBA are increased when a nucleic acid-optimized version of AMBER [44] is employed for these simulations [37].

### 2.3. Structural Characterization of G-Triplex DNA

**Figure 5:**
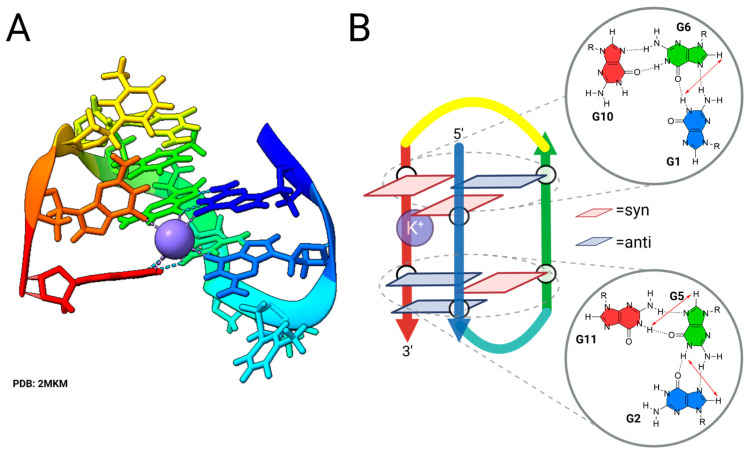
The G-triplex DNA: (**A**) 3D representation of the NMR structure of T1 with bound potassium ion [46]; (**B**) 2D depiction of the antiparallel G3 formed by T1, and the G-triads (in circles) involved in its formation. Red arrows show NOE correlations between H8 and H1 protons of paired bases. Figure created using Biorender.

Although the existence of the telomeric G3 as a folding intermediate had been established through studies of G4-forming DNA oligonucleotides, the propensity of three-G-tract-containing oligonucleotides to form a stable intramolecularly folded G-triplex amenable to direct structural observations was not established until 2013 with the work of Randazzo, Novellino, Bertini and co-workers, who studied the folding of the TBA G4 DNA [45]. Metadynamics (MTD) simulations revealed two intermediate structures involving unfolding of the 3′-terminus of TBA, but MD simulations demonstrated only one of these was stable, which is a G-triplex conformation in which the 3′-terminal G run of the G4 is separated from the remaining core G-triplex. 3′ truncations of the TBA sequence afforded well-folded structures only in the case of T1 (See Table 1). NMR characterization of the structure formed by T1 (Figure 5) revealed a G-triplex structure, and structural models obtained from restrained MD simulation are available as PDB 2MKM (model without bound K^+^) and 2MKO (model with a single bound K^+^ ion) [46]. These structures are stabilized by two G-triads, composed of G1:G6:G10 and G2:G5:G11, with a syn–anti–syn and anti–syn–anti glycosidic bond arrangements, respectively (Figure 5B). The arrangement of the G-triads in the experimentally determined structure is generally in agreement with the computationally determined counterpart, with the exception of G10, which exhibited broader aromatic signals and lacked residual dipolar couplings (RDCs) and nuclear Overhauser effect (NOE) interactions. Nonetheless, when planarity was forced on G10, there were no observable violations of experimental restraints, suggesting the difference is attributable to the lifetime of these structures falling between computational and NMR timescales.

The CD spectrum of T1 is in accord with the structural model, with positive bands at ca. 290 and 250 nm, indicative of *syn–anti* steps for the stacked guanines. The melting behavior of T1 examined by CD and DSC reveals a relative low T_m_ = 33.5 to 34 °C in 70 mM KCl. Thermodynamics demonstrate that the T1 G-triplex is modestly stable at 25 °C (ΔG = −4.0 kJ/mol) due to nearly offsetting favorable enthalpy and unfavorable entropy terms [45,46].

**Figure 6:**
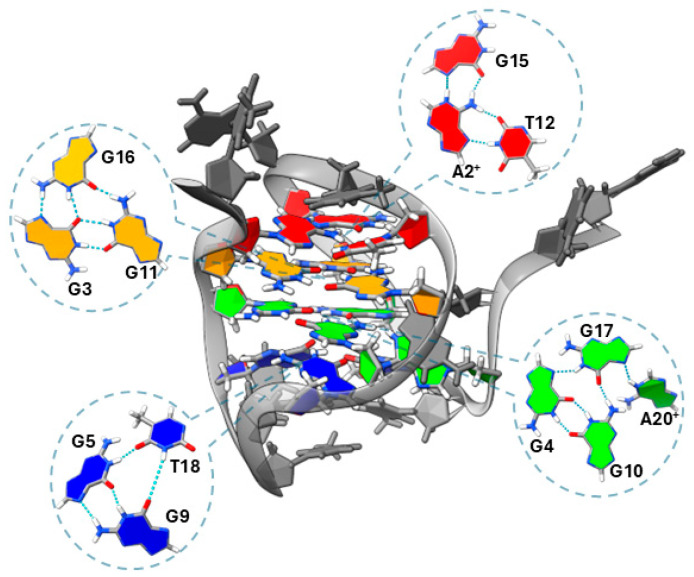
G-triplex adopted by human telomeric sequence htel1 at pH 5 and 5 °C. PDB structure 6TR2 [47]. One G-triad is colored orange, and the second G-triad, which is stabilized by interaction with an adenine base is colored green. These are stacked between a triad involving thymine, protonated adenine, and guanine bases (red) and a GGT triad (blue).

A second experimentally determined structure of G3 DNA comes from studies of pre-folded intermediates of the human telomeric G4. Plavec, Sket and co-workers investigated the pre-folded structures of human telomeric G4 derived from htel1 (Table 1) by NMR [47]. At low pH (5) and temperature (5 °C), htel1 forms a G3 structure in which the 3′-terminal G residues are not associated with the other bases (Figure 6). The G3 contains two G-triads, one of which associates with a protonated adenosine base. These triads are flanked on one face by a triad formed by guanine, thymidine, and a protonated adenosine and on the other face by a GGT triad. The G3 adopts an antiparallel topology and the CD spectrum displays prominent positive peaks at 255 and 300 nm and two negative bands at 235 and 275 nm, commensurate with the syn–anti steps observed for the stacked guanines. An increase in pH to 7 leads to the formation of an antiparallel structure in which all guanine residues’ imino protons are engaged, whereas an increase in temperature from 5 to 20 °C leads to the formation of G-hairpin structure(s).

### 2.4. Further Evidence for G-Triad Formation

**Figure 7:**
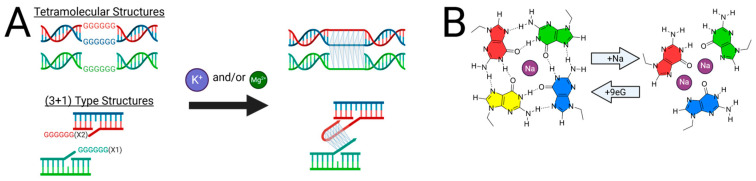
Further experimental evidence of G-triad formation. (**A**) DNA origami setup by Rajendran et al. [48]. (**B**) Interconversion of G-triads and G-tetrads on gold surface proposed by Ding et al. [49]. Figure created using Biorender.

Not long after the first structure of the T1 G-triplex was published, further experimental evidence supported the formation and stabilization of G-triad structures outside of the context of full G3 folding. Rajendran et al. employed a DNA origami framework combined with high-speed atomic force microscopy (HS-AFM) to directly visualize G-triplex intermediates, observing that their formation was influenced by ionic conditions (Figure 7A) [48]. Specifically, their findings suggest that G-triplex assembly is moderately stabilized by both monovalent (K^+^) and divalent (Mg^2+^) cations, with neither ion alone fully optimizing formation. In a similar vein, Ding and colleagues provided experimental evidence of interconversion between G-tetrads and G-triads formed by 9-ethylguanine (9eG) on a gold (Au(111)) surface using scanning tunneling microscopy (STM) and density functional theory (DFT) [49]. Here, structural transitions between motifs were found to be reversible through sequential deposition: adding Na favored triads, while adding 9eG favored tetrads (Figure 7B). Furthermore, G-quartets were homochiral and thermodynamically more stable (binding energy = 1.45 eV/molecule) compared to the heterochiral G-triads (1.33 eV/molecule).

Finally, using a G4 sequence containing a G → T substitution within one of the G-tracts, Heddi et al. solved the NMR structure of a parallel G-quadruplex involving two G-tetrads and one G-triad with a vacant site, revealing a G-triad stabilized by water molecules at the vacant site [50]. Together, these studies highlight that G-triad motifs can arise and be stabilized under diverse environmental conditions and sequence contexts and hint at their potentially broader role in G-rich DNA architectures.

### 2.5. Other Putative G-Triplex Sequences

**Figure 8:**
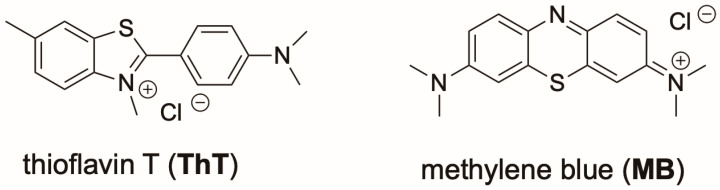
Structure of G3 DNA probes.

In 2018, Zhou et al. proposed a new potassium-stabilized G3 DNA G31 (Table 1) derived from the truncation of the CatG4 aptamer, based on CD, melting analysis, thioflavin T (**ThT**, Figure 8) fluorescence enhancement, and native PAGE [51]. Notably, this new putative G3 DNA features a parallel strand arrangement and a melting temperature (T_m_) of 61 °C, making it the most thermally stable G3 DNA yet reported. Though G31 and derivative sequences with minor alterations have since seen robust use in G3-based biosensing applications (discussed in greater detail in later sections) [52], there has been little further characterization of the structure it forms.

Shortly afterward, Ling-Li Zhao et al. presented a new stable G3 DNA, G3-A (Table 1), that they discovered based on sequential 3′ truncations of the EAD2 G4 DNA [53]. Evidence from electrochemical measurements with methylene blue (**MB**, Figure 8), CD, electrospray ionization mass spectrometry (ESI-MS), and MD simulations were all used to support the claim that it forms a stable parallel G3 DNA with a T_m_ of approximately 62 °C. **MB** both stabilizes this structure and acts as a probe, in which the redox properties of MB are altered upon G3 binding. This enabled the authors to use G3 in an electrochemical aptasensor for cocaine detection.

In our own work, a putative G-triplex-forming region was identified within the cMYC oncogene promoter G4 DNA [54]. G3 DNA formation by cMYCG3 (Table 1) was first assessed through CD and thermal melting analysis, which indicated a parallel strand arrangement and an oligo concentration (5–50 μM)-independent melting temperature of up to 51 °C in calcium-containing buffer. Notably, G3 DNA formation was also tested in a manner that precludes multimer formation using surface plasmon resonance (SPR). Finally, the effect of calcium ions on G3 DNA formation was further tested using a chemiluminescent peroxidase activity assay.

Most recently, Ling-Li Zhao and colleagues proposed a new G3 DNA sequence, which was discovered by systematically optimizing loop composition, sequence length, and flanking bases from G3-A [55]. After initially identifying G3 DNA formation via **ThT** fluorescence enhancement, G3-A was found to bind **ThT** with a higher affinity than any other putative G3 DNA. After verifying monomeric structure formation via native PAGE, CD and melting temperature analysis revealed a parallel topology and melting temperature of 62.5 °C (increased to 74.4 °C with **ThT**) in the presence of potassium. Testing other putative G-triplex sequences, they found there was no correlation between G3 DNA thermal stability and **ThT** fluorescence. One-dimensional ^1^H NMR imino proton spectra (10–12 ppm region) revealed well-defined Hoogsteen hydrogen-bonded guanine signals in the presence of potassium, confirming formation of a hydrogen-bonded G3 DNA.

### 2.6. G-Triplex Formation in RNA

Evidence for RNA G-triplex (rG3) structures has accumulated from several complementary experimental approaches. Wu and co-workers, using smFRET, identified rG3 folding intermediates in the folding of several RNA G-quadruplexes (rG4) from plants (C, AA, ATR, and SMLX, see Table 2) [56]. These experiments revealed that each rG4 folding pathway passes through two discrete intermediates, identified as G-hairpin and G-triplex species. Importantly, the G-triplex intermediate was identified during both folding and helicase-driven unfolding of rG4s. Specifically, using smFRET, these authors identified rG3 intermediates in the unfolding of rG4 structures by DExH1, a plant ortholog of the human DHX36 helicase, which has been shown to resolve rG4 in human cells. More recently, the severe acute respiratory syndrome coronavirus 2 (SARS-CoV-2) nucleocapsid coding sequence element RG1 (Table 2), initially assigned as a parallel rG4 based on CD spectroscopy, UV melting, and thermal difference spectra, was re-evaluated. Detailed analysis of its multiphasic CD melting profile, combined with singular value decomposition, DSC, NMR, and thermal melting analysis and native PAGE of truncations, demonstrated that under physiological conditions RG1 populates a stable parallel RNA G-triplex, likely formed from the 3′ end, rather than a canonical rG4 [57]. In a distinct context, Danyang Ji and colleagues identified an L-RNA aptamer that recognizes the EBNA1 RNA G4 with nanomolar affinity. Structure probing employing selective 2′-hydroxyl acylation with Li^+^-ion primer extension (SHALiPE), CD spectroscopy, electrophoretic mobility shift assays (EMSA), MST, and mutagenesis of specific G-tracts indicates that the loop region of L-Apt1-12 actually forms a G3 that is essential for binding [58]. Furthermore, L-Apt1-12 reduced EBNA1 protein levels in Epstein–Barr virus (EBV)-positive nasopharyngeal carcinoma and gastric cancer cells and showed selective cytotoxicity toward EBV-positive cells but not EBV-negative cells. Collectively, these studies establish that rG3s can exist as stable, physiologically relevant conformations as reproducible intermediates along rG4 folding/unfolding pathways, and as functional motifs conferring selective biological activity. However, compared to the extensive body of work on rG4s (and G3 DNA to a lesser extent), the principles that govern rG3 formation remain largely unexplored. Little is known about how primary sequence context, loop composition, and monovalent cations influence rG3 stability and topology. The limited case studies available do not yet provide generalizable “rules” akin to those established for rG4s. Addressing this knowledge gap will be essential to understand whether rG3s are rare structural curiosities or widespread and tunable regulatory elements in RNA biology.

Computational studies also support the role of RNA G3 in G3 folding. Course-grained and atomistic simulations of the folding of the rG4 TERRA (Table 2) reveal multiple G3 folding intermediates in a multi-process folding pathway [59].

### 2.7. Environmental Conditions and Sequence Contexts That Promote G-Triplex Formation

A complete understanding of G3 and G4 structures requires consideration of the thermodynamic and kinetic landscapes that govern their formation and interconversion. Thermal stability, often represented by the melting temperature, provides a convenient and quantitative measure of structural robustness under given ionic conditions. Typical melting temperature values for intramolecular DNA G4s vary widely, from highly stable parallel forms with T_m_ values upwards of 80 °C (as seen in cMYC promoter G4 sequences, see Table 3) to more flexible hybrid or antiparallel folds such as the human telomeric G4. As demonstrated by the early work on telomeric G3s, these lower-stability G4s are thought to exist in dynamic equilibrium with partially folded intermediates, possibly including G3 species, particularly under sub-saturating cation concentrations. Although the body of work on G3 DNA remains modest compared to the extensive G4 DNA literature, a number of case studies have begun to establish the sequence and environmental conditions that favor its formation. While systematic surveys are still lacking, these individual reports suggest that generalizable “rules” for G3 formation are beginning to take shape, akin to those that have been well established for G4s.

In the case of G4s, cations primarily interact within the central channel formed by G-tetrads in which they coordinate to the O6 carbonyl groups either within the G-tetrad plane or between stacked layers depending on the size and charge of the ion as well as the overall structure of the G4 [60]. In addition to specific binding to G-tetrads, cations enhance G4 stability via charge screening. In terms of the ions relevant to this review, G-quadruplexes have been found to have a stabilization preference following the order of K^+^ > Ca^2+^ > Na^+^ > Mg^2+^ [61].

Like G4s, cation coordination to the O6 carbonyl groups of guanines promotes stability in stacked triads and G3 DNA formation. However, the cationic preferences of G-triplex DNA appear more nuanced and in some cases distinct from G4 DNA. As highlighted in previous sections, early in vitro and single-molecule experiments suggested that like its four-stranded counterpart, the telomeric G-triplex is stabilized by both potassium and sodium [22,28,29]. Though potassium has since demonstrated ubiquitous stabilization of G3 DNA, at similar K^+^ concentrations all G3 structures are less thermally stable than their corresponding full-length G4 structures (Table 3). In most cases the G3 structures melt at temperatures at least 20 °C lower than the parent G4 structures. However, under physiologically relevant intracellular K^+^ concentrations (ca. 100–150 mM) certain G3 DNA structures are still reasonably stable with T_m_ values higher than body temperature (Table 3). In contrast to the case of potassium stabilization of G4 structures, several studies have painted a more nuanced picture for sodium stabilization of G3. The broad NMR peaks and undetectable melting transition by UV for T1 in the presence of sodium indicate that, in contrast to its G4 DNA counterpart, T1 does not form a stable G3 structure in the presence of sodium (Table 3) [46]. Conversely, CD spectroscopy experiments indicated that sodium does promote structure formation in three-G-tract-containing sequences, albeit to a lesser extent than potassium [30]. Most recently, diminished **ThT** fluorescence enhancement, less pronounced CD spectra, and absent or significantly broadened imino resonances of G3-F15 indicate that G3 DNA could not efficiently form in Na^+^-containing solution. Though these differences necessitate further exploration into the physical basis of the difference in potassium- and sodium-stabilized G3 DNAs, Na^+^ clearly has a less pronounced effect in general.

Though experimental evidence is sparse, there is some evidence that NH_4_^+^ has a stabilizing effect on G3 DNA. The ^1^H NMR and 2D ^1^H–^1^H NOESY spectrum of T1 in the presence of NH_4_^+^ resembles its potassium counterpart [46]. However, even in the highest ammonium concentrations a greater proportion of unfolded T1 remained, indicating a slightly weaker effect on G3 DNA stability than potassium. The same trend was observed with G31, which demonstrated slightly lower melting in the presence of NH_4_^+^ compared to potassium (Table 3) [62].

In contrast to G4 DNA, where monovalent ions are the dominant stabilizers, divalent cations can exert unique stabilizing effects on G-triplex DNA. Isothermal titration calorimetry conducted by Jiang and co-workers revealed that K^+^ binding is exothermic, whereas Ca^2+^ binding is endothermic [30]. Furthermore, thermodynamic analyses of thermal melts established a stabilization hierarchy of Ca^2+^ > K^+^ > Mg^2+^ > Na^+^ for three-G-tract-containing G3 DNAs, with Ca^2+^ raising melting temperatures by several degrees (relative to K^+^) in the case of Tel15 (Table 3). This was corroborated by melting temperature analysis of cMYCG3, which exhibited modest stability in K^+^ buffer (T_m_ ~38 °C), which increased to ~51 °C in the presence of only a few millimolar Ca^2+^ (Table 3) [54]. Furthermore, addition of Ca^2+^ enhanced the peroxidase activity of cMYC-G3 in a concentration-dependent manner. Though the universality of this phenomenon needs further study, early evidence suggests that Ca^2+^, which has minimal impact on G4 DNA stabilization, is a uniquely powerful stabilizer of G3 DNA, in some contexts surpassing the effects of potassium.

**Table 3:** Thermal stability of G-triplex-forming sequences and their comparable G-quadruplexes ^1^.

Oligonucleotide	Ion (mM)	T_m_ (°C)	Technique
TBA (G4)	K^+^ (80)	53.0 ± 0.5	DSC [63]
	K^+^ (100)	48.9	UV [30]
	Na^+^ (100)	22.7	UV [30]
	Mg^2+^ (100)	21.2	UV [30]
	Ca^2+^ (100)	54.4	UV [30]
T1 (G3)	K^+^ (80)	33.5 ± 1.0	CD [45]
	K^+^ (100)	26.4	UV [30]
	Na^+^ (100)	undetected	UV [30]
	Mg^2+^ (100)	20.7	UV [30]
	Ca^2+^ (100)	24.2	UV [30]
Hum21 (G4) ^2^	K^+^ (100)	60.1	UV [30]
	Na^+^ (100)	58.4	UV [30]
	Mg^2+^ (100)	39.4	UV [30]
	Ca^2+^ (100)	54.9	UV [30]
Tel15 (G3)	K^+^ (100)	43.2	UV [30]
	K^+^ (110)	40–60 ^3^	CD, DSC [27]
	Na^+^ (100)	32.6	UV [30]
	Mg^2+^ (100)	31.2	UV [30]
	Ca^2+^ (100)	47.7	UV [30]
Tel18 (G3)	Na^+^ (100)	55 ± 1.0	CD [22]
CatG4 (G4)	K^+^ (20)	62.8	CD [64]
G31 (G3)	K^+^ (50)	60	CD [51]
	NH_4_^+^ (300)	51.2 (±0.3)	CD [62]
myc2345 (G4) ^4^	K^+^ (20)	75	CD [65]
cMYC-G3	K^+^ (20)	38	UV [54]
	Ca^2+^ (7.5)	51.0 (±0.7)	UV [54]

^1^ Data for the parent G-quadruplex is in darker color and data for the derived G-triplex is in lighter color. ^2^ Human telomeric G4 sequence GGG TTA GGG TTA GGG TTA GGG. ^3^ Large melting temperature range due to hysteresis in CD melting curves and higher oligonucleotide concentrations in DSC experiments. ^4^ Wild-type cMYC G4 sequence TGA GGG TGG GGA GGG TGG GGA A.

By contrast, magnesium seemingly plays a more modest and cooperative role. For the T1 G3 DNA, the strength of the stabilization effect of Mg^2+^ falls between Na^+^ and Ca^2+^ based on thermodynamic analysis of thermal melts (Table 3) [30]. Notably, their findings also suggest that G3 DNA structure formation is enhanced by the molecular crowding agent PEG 200, in a manner similar to G4 DNA counterparts. Rajendran and colleagues used single-molecule atomic force microscopy (AFM) and smFRET to show that Mg^2+^ alone produced only weak G-triad folding yields in three-stranded constructs [48]. However, when Mg^2+^ was combined with K^+^, yields rose significantly, suggesting that while G3 DNA may not preferentially bind magnesium, there may be a synergistic effect where Mg^2+^ screens backbone repulsion while K^+^ occupies the central ion channel.

Together, these studies highlight a fundamental difference between G3 and G4 DNA: whereas quadruplexes are stabilized by the monovalent ions Na^+^ and K^+^, G-triplex structures are unusually sensitive to divalent ions with Ca^2+^ acting as a direct stabilizer and Mg^2+^ as a cooperative partner. It should be noted that these effects may not be universal given that both Ca^2+^ and Mg^2+^ demonstrated little effect on the CD spectra of G3-F15 [55]. Nonetheless, this divalent dependence warrants further exploration, as it may expand the physiological contexts where G3 DNA can form, particularly in nuclear environments where Ca^2+^ and Mg^2+^ levels fluctuate dynamically.

The primary sequence context of guanine-rich DNA also strongly influences its ability to adopt and stabilize a G-triplex fold in vitro. Perhaps the most critical determinant is the length of its G-tracts, which dictates how many G-triads can stack, and thus its stability. Short G-tracts (e.g., two guanines each) form G3 DNA structures with low thermal stability (Tm < 35 °C, see T1, Table 3) [46]. On the other hand, depending on the stabilizing cation, sequences with G-tracts of three guanines or longer have melting temperatures upwards of 60 °C and can achieve negative ΔG°_37_, indicating that they are stable in physiological conditions [30].

Another consequential aspect of a sequence is the composition of 5′ and 3′ terminal ends and loops, which both have a more nuanced effect on G3 DNA stability as exemplified by the original NMR studies of TBA truncations [46]. Here, 1D ^1^H NMR spectra of successive 3′ truncations of TBA revealed that clear signals corresponding to the characteristic imino protons of G3 DNA only arose upon removal of the last two bases that comprise the 3′-most loop of TBA, and did not fully resolve until removal of the final loop residue. Although this would indicate that terminal G-tracts are critical for G3 DNA stability, subsequent studies have not fit this trend. CD spectroscopy experiments of Tel15 (based on spectra at 0, 6, and 24 h following KCl addition) suggest that the addition of a duplex stem to the 3′ TTA of the G3-forming sequence slows folding speed [33]. Addition of a duplex stem to the 5′ end slows folding speed even more and reduces the proportion of antiparallel structures, according to smFRET experiments. In a similar vein, based on **MB** diffusion current, Zhao et al. found that EAD2 truncations formed the most stable G3 DNA when the 3′-most loop was preserved [53].

There has been some exploration on the general effects of loop sequences on G3 DNA stability. Based on **ThT** fluorescence enhancement, Zhou et al. found that the 5′ TA loop was important for G3 DNA formation of G31, and that short loops favored structure formation in three-G-tract-containing sequences [51]. In sequence optimization studies of G3-A, **ThT** fluorescence enhancement and CD spectroscopy were used to comprehensively study the effect of T/A loops and terminal residues [55]. Ling-Li Zhao and colleagues [55] found the optimal loop composition to be 5′-AA-A-3′, based on a ~2.5-foldfluorescence enhancement relative to other putative G3 DNA sequences. They next found that fluorescence enhancement was at its maximum with a 3′ terminal G-tract. Turning their attention toward the 5′ end they found that a 5′-TC flanking tail had the highest **ThT** enhancement. It should be noted that although these experiments offer some indications on the effect of sequence on G3 DNA stability, the case may be that the results are more reflective of differences in **ThT** binding than stability per se.

Some attention has also been paid to the effect of certain base modifications on G3 DNA stability and folding behavior. Caterino et al. explored the impact of substituting thymidine with 5-hydroxymethyl-2′-deoxyuridine (H), a product of oxidative DNA damage and an epigenetic modification, within Tel15 [27]. CD, DSC and gel electrophoresis analyses revealed that such loop modifications minimally perturbed G-triplex structure and stability, consistent with previous G4 findings. However, the modified sequences exhibited more complex melting behaviors and evidence of intermolecular aggregation.

Zhao et al. systematically studied the effects of 8-methylguanine (m8G) and 8-oxoguanine (oxo8G) substitutions within the T1 G3 [66]. They found that a single m8G incorporation slightly stabilized the G-triplex while tandem m8G substitutions led to a significant ~12 °C increase in melting temperature. Interestingly, when two m8Gs were dispersed rather than adjacent, stability decreased, suggesting that the local structural context and syn/anti conformations of guanine bases critically modulate G-triplex integrity. Similarly, oxo8G substitution modestly stabilized G-triplexes when introduced singly, but had little additive effect when introduced twice.

Finally, Tateishi-Karimata et al. demonstrated that chemical modifications of loop thymidines with tetraethylene glycol (TEG) also influenced G-triplex behavior [67]. Although the primary focus was on G-quadruplex formation, they showed that TEG modifications slightly stabilized G-triplexes and conferred enhanced resistance to nuclease degradation.

Overall, this body of evidence suggests that G3 structures occupy a thermodynamic niche between single-stranded and quadruplex states. In general, G3s display lower T_m_ values than their G4 counterparts, reflecting their reduced stacking interactions and incomplete cation coordination. However, several studies suggest that G3 DNA structures fold from unstructured DNA faster [21,28,39,68]. This kinetic lability may endow G3s with distinct biological roles, such as acting as transient folding intermediates or regulatory conformations that can respond rapidly to local environmental fluctuations, particularly in cases where the overall stability of G3 and G4 DNA differs only slightly, as in the case of telomeres. The dynamic interchange between G3 and G4 forms thus represents a key target of nucleic acid regulation that couples the thermodynamic hierarchy of guanine-rich structures with their capacity for structural plasticity in vivo.

### 2.8. Structural Polymorphism and Challenges in the Study of G-Triplex Structures

**Figure 9:**
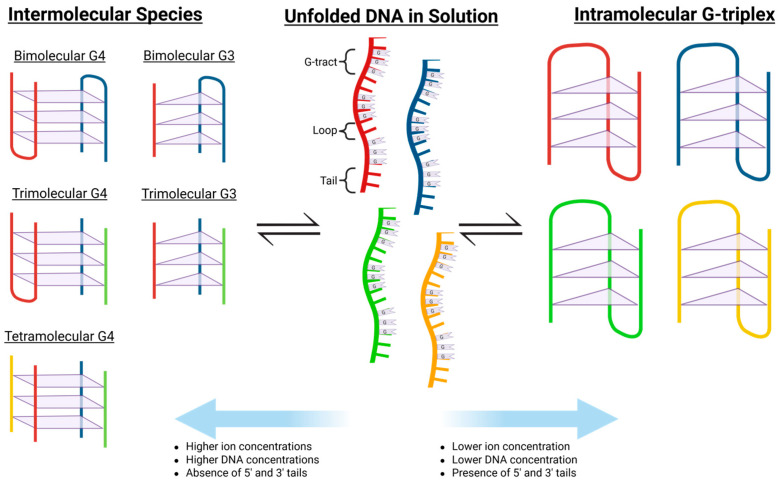
Competition between intramolecular and intermolecular structure formation. Conditions favoring intermolecular or intramolecular species are shown at bottom. Figure created using Biorender.

When in solution, many guanine-rich oligonucleotides have the propensity to form an equilibrium mixture of intramolecularly folded and multimeric species (Figure 9) [69]. Of course, this phenomenon extends to putative G-triplex sequences and adds a significant challenge to identifying bonafide G-triplex-forming sequences. In the case of G4 sequences, it has been demonstrated that intermolecular species formation is favored by terminal guanine residues, higher ion concentrations, and higher oligonucleotide concentrations [10]. Thus, these are important considerations when studying putative G3 DNA sequences. Several examples in the literature highlight this.

In the case of telomeric G3 sequences, early work using Tel21 in buffer containing 100 mM Na^+^ verified intramolecular structure formation using multi-concentration melts [22]. However, subsequent CD melting, DSC, and native PAGE experiments using Tel15 in 100 mM KCl indicated intermolecular structure formation [27]. Though this difference may be attributable to the ions used in each study, another cause could be the presence of the 5′ TTA “tail” portion.

In another example, shortly after the establishment of G31 as a stable G3 DNA-forming sequence, additional experimental evidence suggested that G31 may actually form a parallel, tetramolecular G4. This conclusion was based on observed two-staged melting behavior, concentration-dependent melting temperatures, and fluorescence behavior of 5′ pyrene-modified G31. In addition, G31 displays similar fluorescence enhancement and binding thermodynamics with auramine O compared to the G4 CatG4 [69]. This was directly contradictory to the evidence of intramolecular G3 DNA formation. However, given that potassium concentrations in these latter studies were twice that of the initial G31 study these differences may be attributable to higher ion concentrations promoting intermolecular association of strands due to charge shielding. Nonetheless, these contradictions highlight the necessity of thorough experimental evidence that precludes the possibility of multimer formation. Over reliance on techniques that do not differentiate intramolecularly bonded G3 DNA from intermolecular G4 DNA, such as CD spectroscopy and fluorescent probe enhancement techniques, can lead to faulty conclusions around structure formation.

## 3. Biological Functions of G-Triplexes

### 3.1. G-Triplex and Regulatory Proteins

One of the most potent hints at the evolutionary importance of G4 DNA is the existence of G4 binding proteins and helicases. One such example is Pif1 helicase, whose G4 unwinding activity is essential for replication progression through lagging strand G4s [70]. Interestingly, smFRET-based examination of the mechanism by which Pif1 unwinds G4 DNA revealed that it repetitively unfolds G4s to ssDNA through a G3 intermediate [71]. Furthermore, these experiments indicate that Pif1 binds to G3 with higher affinity than the corresponding G4, hinting at the biological significance of G3 DNA.

Another example of a functionally important G4 binding protein is breast cancer type 2 susceptibility protein (BRCA2). Mutation of BRCA2 results in susceptibility to a variety of cancers, particular those of the breast and ovaries. The molecular basis of this correlation appears to be BRCA2′s role in regulating telomere replication and length [72]. Recently, Lee and colleagues elucidated the mechanism behind this phenomenon. Using smFRET and EMSA, they demonstrated that BRCA2 interacts with telomeres through binding G3 intermediates [73]. Upon G3 binding, BRCA2 prevents degradation of G4-stalled replication forks and facilitates the restart of replication. The interactions of BRCA2 with telomeric structures was reviewed in further detail by the same group [74].

One of the early breakthroughs demonstrating the ubiquitous existence of G4 DNA in vivo utilized an engineered, G4 structure-specific antibody (BG4) to quantitatively visualize G4 DNA formation in human cells [75]. Recently, enzyme-linked immunosorbent assay (ELISA) and single-molecule pull-down (SiMPull) experiments revealed that BG4 binds to TelG3 with similar affinity to 8-aza-7-deazaguanine-mutated TelG4, with a K_d_ ~10 nM [76]. The revelation that BG4 also binds to G4 DNA folding intermediates like the G-triplex complicates the analysis of data obtained with this antibody.

Two studies have provided evidence that G-triplexes can function in vivo in *Escherichia coli* (*E. coli*). Abram et al. used green fluorescent protein (GFP) reporter assays to test whether insertion of T1 into the antisense strand of the σ70 promoter region could modulate transcription [77]. They found that, unlike parallel or antiparallel G4s that suppressed expression, the G3 motif had little effect on GFP output, suggesting that in a promoter context G3 formation does not strongly interfere with RNA polymerase binding or activity, at least in the case of the relatively unstable T1. Similar experiments with more stable G3 DNA-forming sequences have demonstrated otherwise. Teng et al. scanned the *E. coli* genome and identified nearly 200 potential three-layer G3 motifs and 28 four-layer motifs [78]. Using smFRET, they showed that DNA polymerase I can readily replicate through a potassium-stabilized three-layer G3 DNA but is significantly impeded by the more stable four-layer form, establishing that G-triplexes can act as real barriers to replication in bacterial cells. Together, these results illustrate that G-triplexes can be stable and biologically consequential structures in vivo, though their functional impact depends heavily on genomic context and length of G-tracts.

Li et al. [79] demonstrated that G3 DNA can serve as effective substrates for Clustered Regularly Interspaced Short Palindromic Repeats (CRISPR)-Cas12a trans-cleavage activity. Using multiple orthogonal methods (FRET, circular dichroism, PAGE, and NMR), they showed that the thrombin-binding aptamer truncation T1 and a telomeric G3 structure were rapidly degraded upon Cas12a activation with complementary target DNA, confirming structural disruption of these motifs. Kinetic analyses revealed that cleavage rates were slower for Na^+^- and K^+^-stabilized Tel21-G3, even slower for TBA, and slowest for Tel22 [79].

### 3.2. Interaction of Ligands with the G-Triplex

**Figure 10:**
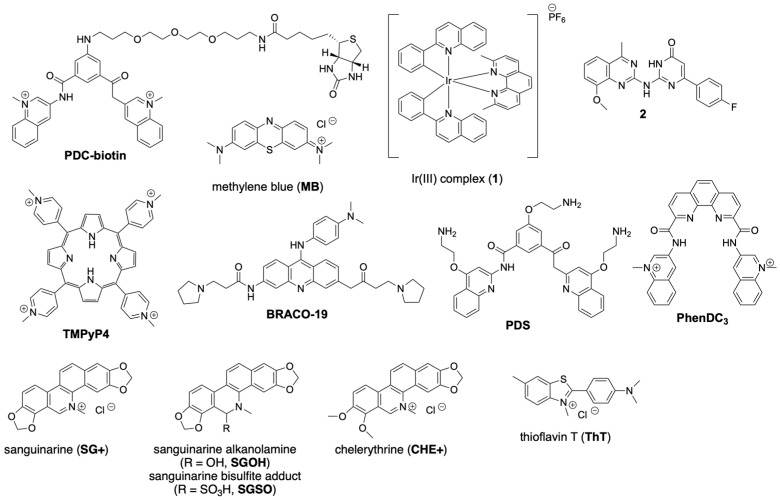
Structures of small-molecule ligands reported to bind to G3 DNA.

The binding of small molecules to G3 DNA has emerged as a key area for understanding their stability and functional potential. Early work demonstrated that classical G4 ligands can also target G3 structures. Using DNA origami and AFM imaging, Sugiyama et al. found that the G4 binding ligand bisquinolinium pyridine dicarboxamide (**PDC-biotin**, Figure 10) may bind to and promote structure formation of G-hairpin and G-triplex DNA [80]. Though the constraints of the DNA origami methods do not necessarily translate to the intramolecularly folded G3 targets within the human genome, these results served as an initial indication that traditional G4 ligands may indiscriminately bind to G-tetrad and G-triad-based non-canonical DNA structures. At about the same time, Ma and co-workers reported an Ir(III) complex **1** (Figure 10) that, upon binding to folded G3 or G4 DNA, exhibited enhanced luminescence; however, no thermodynamic binding data for 1 was reported [81]. A subsequent screening of potential G3 binding compounds identified a dihydropyrimidin-4-one derivative **2** (Figure 10) that was capable of stabilizing the 13-mer TBA G3 (TBA13, Table 1) [82]. Interestingly, this compound was also found to stabilize parallel G4 DNA as well, but not hybrid and anti-parallel G4 DNA. Bio-layer interferometry (BLI) and SPR experiments utilized a peptide scaffold to assemble G3 and G4 constructs from G-hairpin oligonucleotides and adjacent single-stranded oligonucleotides (G3 mimic) or adjacent G-hairpin oligonucleotides (G4 mimic) to assess the promiscuity of several prominent G4 ligands [83]. Here, it was found that the G4 binding ligands **TMPyP4**, **BRACO-19**, **PDS**, and **PhenDC3** (Figure 10) bind to the G3 construct with similar affinity and kinetics as the G4 construct.

An additional study highlighted **MB** as a small molecule with unexpected G3 selectivity. Zhao and co-workers reported that G3-A (Table 1) formed a stable G3 DNA whose affinity for **MB** was marginally stronger (K_a_ = 6.1 × 10^5^ M^−1^) than that of the corresponding G4 DNA EAD2 (K_a_ = 4.8 × 10^5^ M^−1^) [53]. Electrochemical assays showed that G3-A reduced the redox current of **MB** more significantly than EAD2, consistent with tighter binding. CD melts further revealed that **MB** raised the T_m_ of G3 by ~18 °C, indicating substantial stabilization. ESI-MS confirmed a 1:1 noncovalent complex, while molecular dynamics simulations suggested that **MB** intercalates into the triad planes of G3-A, in contrast to its external stacking on the G4 EAD2. This altered binding enabled use of the complex as an electrochemical signal element, constructing a cocaine aptasensor with improved sensitivity compared to analogous G4-based systems. Together, these findings position **MB** as a promising G3 DNA ligand with both stabilizing and biosensing applications.

More recently, ligands have been exploited to exert external control over G3 assembly. This work is based on the pH-dependent equilibrium of aqueous solutions of the benzo[c]phenanthridine alkaloid sanguinarine, which favors a planar, cationic form (**SG+**) at low pH and a non-planar, alkanolamine form (**SGOH**) at high pH (Figure 10). Gao et al. identified a pH-triggered G-triplex switch that can function in the presence of K^+^ ions through the conversion of sanguinarine from its G-triplex stabilizing iminium form (**SG+**) at low pH to its non-binding alkanolamine form at high pH (**SGOH**) [84]. The addition of SG to the otherwise unfolded TBA13 at pH 4.9 results in a CD spectrum characteristic of an antiparallel G3. ITC experiments confirmed the binding affinity of **SG+** to TBA13 of approximately 3.5 × 10^5^ M^−1^ with a stoichiometry of 1:1. Similar binding stoichiometry and higher affinity were determined for **SG+** binding to the G4 TBA (6 × 10^5^ M^−1^). At a pH of 8.6, however, **SGOH** does not bind to TBA13 and no induced structure formation occurs. Similarly, the related alkaloid chelerythrine was also reported to bind to G3 TBA13 only under low-pH conditions favoring the cationic, planar form **CHE+** (Figure 10), with little difference in binding affinity when comparing **SG+** to **CHE+**. Employing this pH-dependent behavior, these workers demonstrated reversible switching of TBA13 G3 formation through pH changes, including using the photoacid ethyl p-toluene sulfonate (ETS) to enable photo switching between single-stranded and G3 forms in the presence of sanguinarine. In a related approach, these workers showed a similar switch between G3 and single-stranded forms of TBA13 under redox cycling involving the formation of **SGSO** (Figure 10) the bisulfite addition product of **SG+** in the presence of Na_2_SO_3_ and its conversion back to **SG+** in the presence of H_2_O_2_. In contrast to the relatively unstable G3 TBA15, this same group found that **SG+** binds to the more stable T1 G3 with 1:2 stoichiometry, suggesting that the formation of T1 dimers and reversible switching of **SG+** induced dimeric nano assemblies of T1 through pH and redox reactions [85].

Thioflavin T (**ThT**) (Figure 8) has emerged as one of the most widely used ligands for G3 DNA, based upon its enhanced fluorescence in the presence of G3. Hui Zhou et al. identified that G3 G31 (Table 1) increases **ThT** fluorescence, similar to the enhancement observed in the presence of G4 DNA [51]. Further work by Ling-Li Zhao and colleagues identified an optimal sequence, G3-F15 (Table 1), that increases **ThT** fluorescence up to 3200-fold, representing the strongest signal enhancement reported for any G-rich sequence [55]. Fluorescence titrations established a micromolar-affinity, 1:1 G3–**ThT** complex (K_a_ ≈ 6.1 × 10^5^ M^−1^), which compares well to ThT binding to G4 DNAs (K_a_ ≈ 0.3–2.5 × 10^5^ M^−1^) [86]. CD and NMR confirmed that a parallel G3 fold further stabilized upon dye binding, which they leveraged to design a label-free biosensor for uracil-DNA glycosylase activity, demonstrating the utility of G3 DNA–ligand complexes in enzyme activity detection. Expanding on the effect of primary sequence context showed that duplex DNA adjacent to the 5′ end of a G3 promotes parallel folding and significantly enhances **ThT** fluorescence, with 2–3 base spacers between the duplex and G3 yielding the highest signal [87]. By contrast, dsDNA positioned at the 3′ end favored antiparallel conformations and weaker fluorescence. These findings establish **ThT** as potential reporter of G3 DNA formation and a versatile platform for biosensing, while highlighting how sequence context and duplex adjacency may affect probe association with G3 DNA.

In response to the difficulties surrounding lack of selectively with small-molecule ligands, some attention has shifted toward the development of oligonucleotide-based ligands. Wen and colleagues used this approach to target G3 sequences by combining a three-guanine PNA (peptide nucleic acid) oligonucleotide with a 23-amino-acid portion from the G4 binding domain of RNA helicase associated with AU-rich element (RHAU) [88]. This conjugate targeted G3 sequences with up to 1000 times the binding affinity of traditional small-molecule ligands and was shown to inhibit DNA motor protein progression upon binding.

Together, these studies underscore that G3 DNA is broadly accessible to small-molecule recognition, whether through the promiscuity of classical G4 ligands or the more functionally selective ligands like **MB** and planar alkaloids such as sanguinarine. The rapid adoption of **ThT** as a light-up probe has further cemented G3 DNA as a functional moiety for biosensing applications, while the emergence of oligonucleotide–peptide hybrids illustrates how ligand design is beginning to move beyond repurposed G4 DNA binding ligands. A central challenge remains the development of ligands with genuine specificity for G3 over G4, as most current examples bind both structures. Future progress will likely come from integrating structural insights into G3 DNA folding with rational ligand design, enabling both the probing of G-triplex formation and the translation of these motifs into robust diagnostic and therapeutic platforms.

### 3.3. Catalytic Activity of G3 DNA

Although G3 DNAs were first recognized as structural intermediates of G-quadruplexes, accumulating evidence shows they can act as functional catalysts in the presence of appropriate metal ion cofactors or coenzymes. One of the earliest demonstrations came from asymmetric catalysis: Xu et al. showed that a Cu(II)–T1 complex significantly accelerated Diels–Alder cycloadditions, achieving full conversion and up to 51% enantioselectivity, thereby highlighting the potential of G3 DNA as a metallo-DNAzyme for enantioselective organic synthesis [89]. In a different context, a truncated G3T1 sequence that forms a parallel monomolecular G3 DNA can function in quadruplex-templated ligation, catalyzing strand ligation with high efficiency even under harsh conditions, thus extending the repertoire of nucleic acid catalysis beyond duplex- or G4-templated reactions [90].

Among these activities, however, the best-characterized catalytic role of G3 DNA is its association with the iron porphyrin hemin to function as a peroxidase DNAzyme. Shortly after the NMR structure of T1 was resolved, Wang and colleagues demonstrated that T1, much like many G4 DNAs, can bind hemin to form a G3–hemin complex capable of catalyzing the oxidation of 2,2′-azino-bis(3-ethylbenzothiazoline-6-sulfonic acid) (ABTS) by hydrogen peroxide [91]. The catalytic efficiency was comparable to that of G4–hemin complexes, and crucially dependent on the integrity of the G3 fold, as single-guanine mutations abrogated activity. Moreover, the G3–hemin system generated robust and long-lasting colorimetric signals, making it particularly promising for biosensing applications.

Taken together, these findings suggest that G-triplexes are more than transient folding intermediates or targetable structures: they can support diverse catalytic functions, from asymmetric synthesis to nucleic acid ligation, with peroxidase activity standing out as the most thoroughly explored function. This latter function provides a natural conceptual bridge to the application of G3-based DNAzymes in biosensor development.

## 4. Applications of G-Triplexes

### 4.1. G-Triplex Function in Biosensor Development

**Figure 11:**
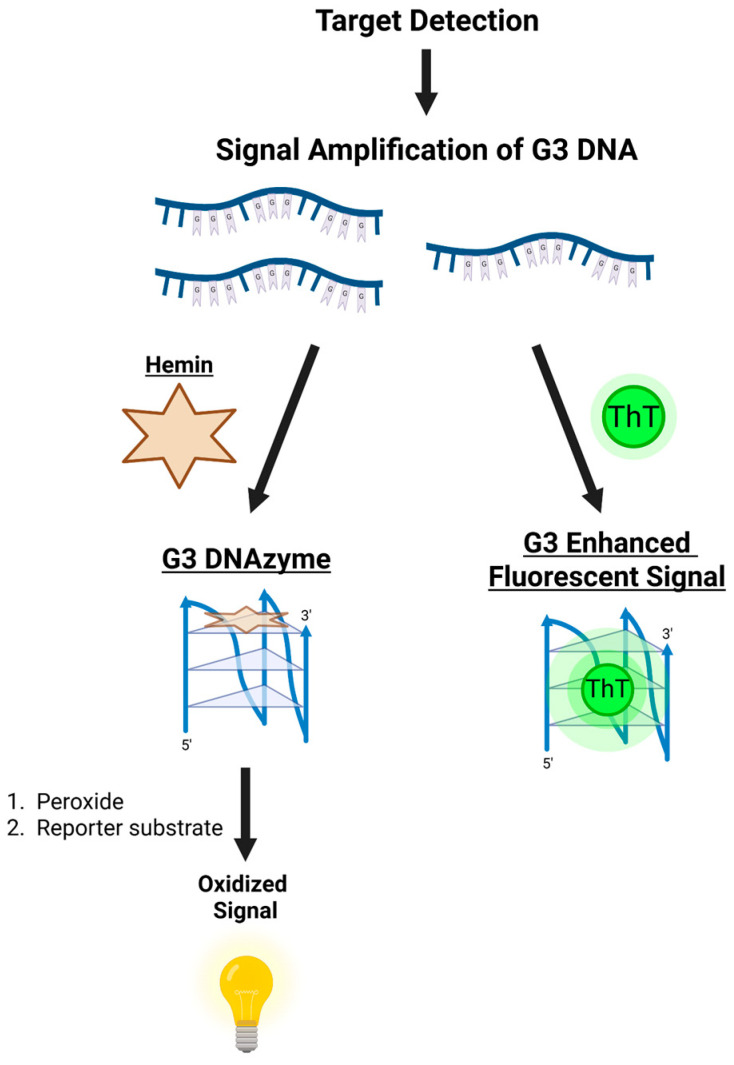
The G-triplex’s function in biosensing assays. Two major signaling modalities are shown: hemin-dependent peroxidase reporting (**left**) and turn-on fluorescence in the presence of **ThT** (**right**). Figure created using Biorender.

In addition to their diverse genomic function, G-quadruplexes have also garnered attention as chemically functional molecules. One example of their functionality is their capacity to serve as catalytic DNAzymes. Through binding hemin, G4s exhibit peroxidase activity, which can be quantified through colorimetric or luminescent measurements. G4 peroxidase-based assays of this nature have been applied toward sensitive detection of free radicals, specific DNA sequences, metal ions, small molecules like cocaine, and proteins [92]. Since the first structural characterization of the T1, the G-triplex has received attention for its similar capacity as a biosensor (Figure 11). Addition of T1 G3 to hemin was shown to increase the intrinsic peroxidase activity of hemin in a similar capacity to the equivalent G4, but with an extended duration [91]. Much later, Jielin Chen and colleagues found that G3T1 has lower peroxidase activity than equivalent G4, which can be recovered with addition of TGGGT to form a (3 + 1) G-quadruplex [93]. Several biosensors have since been developed based on G3–hemin peroxidase activity.

Another approach to G3 biosensors has centered around their ability to bind to probe molecules with luminescent or fluorescent properties to alter the signal output of the probe. The earliest approach of this strategy utilized the luminescent Ir(III) complex **1** (Figure 10) that, upon binding to folded G3 or G4 DNA, exhibited enhanced luminescence compared to ssDNA and dsDNA [81]. Following digestion of a G3 complementary oligonucleotide with mung bean nuclease, the signal generated by addition of this luminescent “switch-on” probe indicated the nuclease’s activity in a sensitive and selective manner. More recently, **ThT** has been a popular choice in developing a variety of fluorescence switch-on assays. Below, we will discuss how the G-triplex has been utilized in DNAzyme and fluorescent ligand-based biosensors for the detection of a variety of different targets.

Though G3 DNA is one essential feature of the biosensors discussed herein, perhaps just as important for most assays is the amplification strategy used to boost signal strength. Unfortunately, a more detailed discussion of the topic of biosensor amplification strategies is beyond the purview of this review. For a more robust discussion of effective strategies, several other reviews have been written on the topic [94,95,96].

### 4.2. G-Triplex Nucleic Acid Detection Assays

Generally, the use of G3 DNA nucleic acid detection assays centers around the increased flexibility in sensor design as compared to designs that incorporate G4 DNA. Most designs are based on the use of hairpin probes meant to hybridize with a complementary target. Therefore, the requirement of fewer G:C base pairs to lock G3 bases into a hairpin structure when a target is not present means decreased hairpin stability compared to G4 and higher hybridization with the target nucleic acid.

As the first group to exploit the ability of G3 DNA to serve as a peroxidase-mimicking DNAzyme in a bioassay, Tian, Zhou and co-workers utilized G31 to catalyze the oxidation of Amplex Rex by H_2_O_2_ to generate a fluorescent signal [62]. Utilizing this, they developed an assay for microRNA (miRNA) detection based on the hybridization of target miRNA to a G3 DNA-containing probe and cleavage by double-stranded specific nuclease (DSN). The assay demonstrated a detection limit of 2.0 picomolar and good specificity towards the target microRNA.

Utilizing the **ThT**:G3 interaction, Zhou et al. devised a hairpin-based miRNA detection assay, in which G-tracts locked within the hairpin stem are released upon miRNA binding to the recognition region of the loop [51]. Compared to the G4 equivalent, the G3-based molecular beacon requires less stem G:C base pairs (using G-tract) to outcompete G3 formation, and consequently is slightly more sensitive, though within one order of magnitude, compared to the equivalent G4-based molecular beacon. They further devised a strand displacement amplification strategy, using template composed of a 3′ complementary target recognition region, a 5′ G3 coding region for signal production, and a nicking site in between. Upon hybridization of target miRNA to the 5′ region, polymerase extends the target along the template to synthesize the nicking site and G3 DNA sequence. Upon nicking, polymerase may act on the new active site and displace the previously synthesized G3 DNA sequence to make a new one. Once released, the G3 DNA sequence may fold and bind **ThT** to generate a fluorescence signal. Comparison of G3 DNA and G4 DNA in the same assay indicated that G3 DNA induces more **ThT** fluorescence and has higher miRNA sensitivity [51]. In more recent work, this group combined the use of a G3-based molecular beacon with duplex-specific nuclease signal amplification (DSNA) to afford a 500-fold enhancement in miRNA sensitivity and effective discrimination between targets with only a single base mismatched. Importantly, the authors attribute this selectivity to the advantages of using a G3-based molecular beacon, which requires a shorter stem compared to G4-based molecular beacon, making it more compatible with DSNA [97]. A variety of other G3 DNA-based biosensors have been devised to detect miRNAs both in vitro and from cell lysate samples. Chen and colleagues developed an enzyme-free biosensor for miRNA-21 detection using G31: **ThT**, silver nanocluster pairs, and a dual hairpin amplification strategy [98]. Their biosensor had a detectable range of 0.1–300 nM and a limit of detection (LOD) of 67 pM, could discriminate between miRNA-21 and similar miRNAs, and was functional with cell lysates. Zhou and co-workers describe a G3 biosensor exploiting **MB** binding and isothermal exponential amplification (EXPAR) for an electrochemical biosensor combined with the EXPAR strategy for sensitive and selective electrochemical detection of miRNA let-7A [99]. This biosensor has a detection range of 1 fM–10 nM and LOD of 0.45 fM. This assay could differentiate single-base mismatches and was validated in A549 lung cancer cell lysates. The group of Wang, Zhang, and co-workers also employed the ability of G3 to sequester **MB** and thus decrease diffusion current in a λ-exonuclease amplification strategy for miRNA-141 detection, with a detection range of 100 fM to 1 nM and LOD of 16 fM [100]. These workers further demonstrated that the assay can discriminate between single-base mismatches and can detect 10 pM of miRNA-141 in serum with a high recovery rate. Xu and co-workers employed dimeric G3 and **ThT** fluorescence in a dual-mode interactive strand displacement assay for miRNA-155 [101]. This assay achieves a linear detection range of 10 fM to 1 nM with an LOD of 1.06 fM. Wang and co-workers developed a biosensor integrating a molecular beacon-like catalyzed hairpin assembly circuit with Au-based metal–organic framework carriers loaded with G-triplex/hemin DNAzymes, supported on conductive g-C_3_N_4_ nanosheets, to couple nucleic acid amplification with catalytic signal generation [102]. The synergy of the amplification, high-density nanozyme loading, and optimized electrode interface enabled highly sensitive electrochemical detection of miRNA-721, a biomarker of acute myocarditis. The system achieves a detection limit as low as 0.25 fM, a broad linear range (0.5 fM–1 nM), excellent specificity down to single-base mismatches, and strong stability/repeatability, making it promising for clinical applications.

Another nucleic acid target of interest for G3 biosensors has been human immunodeficiency virus (HIV) DNA. Ruiying Li and colleagues were the first to develop a biosensor platform for HIV DNA detection, utilizing G3 peroxidase activity and an isothermal exponential amplification reaction strategy [103]. In this assay, ssDNA probes with portions containing the complementary sequence to target HIV DNA, an antisense sequence of a nicking enzyme recognition site, and a G3 complementary sequence were created. Upon hybridization with target DNA, short G3 oligonucleotides are generated exponentially through polymerase and nicking enzyme-driven amplification cycles. The newly generated G3 products were then combined with hemin to catalyze the oxidation of ATBS in the presence of H_2_O_2_. The colorimetric signal produced by this reaction could detect as little as 4.7 femtomolar DNA. The same group then utilized G3-A peroxidase activity for miRNA let-7a detection using a rolling-circle amplification (RCA) strategy [104]. Upon hybridization of target miRNA with a padlock DNA probe, the probe is ligated and circularized by T4 RNA ligase 2, then replicated with phi29 DNA polymerase. The G3-forming portions of newly synthesized DNA may then fold, bind hemin, and catalyze the ABTS oxidation reaction. As before, the colorimetric signal produced can be measured to detect as little as 37 femtomolar miRNA, compared to the detection limit of 1 picomolar using a G4 equivalent. Furthermore, the assay has an analytical range of 0.1–100 picomolar miRNA and can distinguish single-nucleotide differences from target miRNA.

Cia and colleagues developed an HIV biosensor utilizing the G31: **ThT** interaction and a catalyzed hairpin assembly amplification strategy [105]. Upon optimization, this assay could detect as little as 33 pM of HIV DNA oligonucleotide and was capable of discriminating 1–3-base mismatches. Yi and colleagues developed an HIV DNA biosensor utilizing the G31: **ThT** interaction and a dual-template, multi-cycle, nicking enzyme amplification strategy [106]. This assay had a detection range of 50 fM–2 nM and an LOD of 30.95 fM, was selective against single-base mismatches, and could detect HIV DNA in serum down to 100 fM with high recovery rates.

Jin and co-workers report the detection of HIV DNA using G3-catalyzed oxidation of luminol and a cyclic strand-displacement amplification strategy [107]. Using a portable luminometer for point-of-care testing, this sensor had a linear range of 0.05–10 nM and an LOD of 29.0 pM and could specifically discriminate between single-base mismatch oligonucleotides. The assay was also able to detect an HIV-DNA oligonucleotide in human serum with high recovery rates.

There has also been interest in employing G3-based biosensors in the detection of p53 DNA. Yan and co-authors described a hairpin probe containing G3 complement combined with the EXPAR strategy and **ThT** to develop a sensitive assay for p53 DNA detection, with a dynamic range of 10 pM-300 nM of DNA, single-base mismatch discrimination, and the ability to perform detection in spiked serum samples [108]. By careful optimization of the G3 molecular beacon structure, Li and co-workers demonstrated a biosensor for p53 DNA with a detection limit of approximately 0.3 nM, well below the limit of the equivalent G4-based molecular beacon and traditional dual-labeled fluorescent molecular beacons [109].

Other studies have focused on the detection of viral DNA, including in clinical samples. Li and Liu report a G3-based reporter for CRISPR-Cas12a in a highly sensitive detection assay for human papilloma virus (HPV) DNA. Utilizing trans-cleavage of a fluorescently labeled G3 T1 DNA, these authors demonstrate sensitive detection of SARS-CoV and SARS-CoV-2 N-gene and HPV16/18 L1-gene DNA. Detection limits reached 50 pM for unamplified DNA and 0.1 aM for PCR-amplified DNA [79]. The assay was further utilized to test clinical samples for HPV16+/18+ after PCR amplification and had 100% positive predictive agreement and 97.5% negative predictive agreement, detecting 94.7% of HPV-positive clinical samples without a single false positive. A related CRISPR-Cas12a approach using a TBA11 fluorescent probe was reported for the detection of monkeypox and HPV DNA [110]. Upon optimizing conditions to promote simultaneous G3 formation and Cas12a cleavage, these workers found that the G3-based assay could detect as little as 38.7 fM of monkeypox viral DNA, an order of magnitude lower than the ss-DNA equivalent assay. They further validated the assay by testing monkeypox viral DNA-spiked serum samples and testing cervical swab samples for HPV viral DNA.

G3-based biosensors have also been employed with other amplification strategies to detect target DNA. In an optimized entropy-driven circuit amplification strategy, Liu and co-authors harnessed the enhanced fluorescence of **ThT** in the presence of G3 to detect target DNA in an enzyme- and label-free manner [111]. This assay could detect concentration ranges of 10 pM to 100 nM DNA with an LOD of 3.4 pM. The assay required less detection time than comparable methods, could differentiate single-base mismatches, and could perform detection in DNA serum with high recovery rates.

Noting that the addition of double-stranded DNA on the end of G31 leads to increased **ThT** fluorescence that is proportional to the dsDNA length, Wang and co-authors devised a method for detection of the transcription terminator of the nopaline synthase (NOS) gene with a detection range of 0–200 nM and an LOD of 2.22 nM [112]. This assay could discriminate against DNA with two or more mismatches and was used to detect NOS terminator DNA in soybean extracts.

A novel method for detection of antibiotic resistance genes was developed by integrating mDNA-Au@Fe_3_O_4_ and a DNA concatemer with fluorescence due to G31: **ThT** interaction [113]. Tang and others report a detection range of 0.1 to 30 nM and an LOD of 62.4 pM, while the assay could be applied to test isolated bacterial samples. You and co-authors describe a biosensor for *Mycoplasma ovine pneumonia* DNA detection that combines a G-triplex DNA probe with a metal–organic framework (MOF)-functionalized electrode surface [114]. This assay had a linear range of 0.1 to 1000 nM and an LOD of 1.00 pM, could maintain performance in 10% goat serum, and was regeneratable.

Applications of G3-based biosensors for DNA damage have also been reported. Dong and co-authors describe a biosensor for 8-oxo-7,8-dihydroguanine (8-OG) devised using G3-F15, **ThT**, and a nicking endonuclease-mediated rolling-circle amplification strategy [115]. The assay had a linear range of 1–500 amol and an LOD of 0.18 amol. This assay could determine locus-specific 8-OG accurately at low abundance and could be used to test cell DNA extracts for 8-OG.

Finally, G3-based biosensors can also be applied to the detection of RNA. A study using Htel as the G3 to detect SARS-CoV 2 mRNA had a detection limit as low as 10 pM [116].

### 4.3. G-Triplex Protein Detection and Enzyme Activity Assays

G3 biosensors have also been utilized to detect the presence of proteins and activity of enzymes. Two closely related label-free fluorescence strategies used streptavidin–biotin as a model to demonstrate G3 reporting with exonuclease III (Exo III) amplification. Liu and co-workers coupled a biotinylated binding probe to an Exo III-driven recycling loop that liberates many G3 segments to bind **ThT**, achieving 5.6 pg/mL limits and strong selectivity across non-targets [117]. In an inversion of this design, Xue et al. utilized an assay in which a 3′-biotin–streptavidin complex sterically protects the hairpin from Exo III [118]. In this “signal-off” variant, streptavidin binding suppresses G3 release and **ThT** activation; compared to a G4 beacon, the biosensor reached optimal readout with less enzyme and shorter reaction time due to the more concise G3 sequence constraints.

G3 DNA peroxidase functionality has been exploited for sensitive and selective detection of endonuclease activity and inhibition [119]. Here, a dsDNA substrate composed of a G3-forming region, an enzyme recognition region, and a G3 complementary region was utilized to detect restriction enzyme activity of *Escherichia coli* RY13 (EcoRI). When combined with EcoRI, the substrate was cleaved into its constituent components, which in turn separated into single strands and allowed G3 formation. Upon association with hemin, the colorimetric signal generated by 3,3′,5,5′-tetramethylbenzidine (TMB) oxidation byH_2_O_2_ was several times higher with EcoRI than in its absence, or in the presence of an EcoRI inhibitor. Furthermore, the signal generated by G3 exceeded that of G4s tested in the same assay conditions, likely due to its comparable instability in both folded and hybridized forms.

In another G3: **ThT**-based enzyme detection assay, DNA adenine methyltransferase (Dam MTase) activity was quantified using a dumbbell-like G3 molecular beacon and RCA [120]. A biosensor for alkaline phosphatase activity was developed using G3-A, **ThT**, and a dumbbell-shaped probe/Exo III detection strategy [121]. The assay had a linear range of 0.00002–0.002 U/µL and an LOD of 1.8 E-5 U/µL, was discriminant against other enzymes, and was capable of performing detection in serum with high recovery

In an example of employing G3 DNAs for detecting pathogens, Pang et al. developed a biosensor for *E. coli* O157:H7 detection using G31′ peroxidase activity and an Exo III-assisted amplification strategy [122]. The assay had a linear range of 1.3 × 10^3^–1.3 × 10^7^ CFU/mL and an LOD of 1.3 × 10^4^ CFU/mL, was selective against other foodborne pathogens, and could detect as little as 2.3 × 10^3^ CFU/mL in spiked milk samples.

Finally, G3-based biosensors have even been utilized to develop a point-of-care testing device for cardiac troponin I detection by integrating the telomeric G3 into a carbon nanotube-based field effect transistor [123]. The sensor had an LOD of 0.33 fg/mL (12× better than ssDNA reporter), was discriminant against other proteins, was capable of detection in plasma samples, and was successfully utilized in a portable device for rapid detection in clinical samples.

### 4.4. G-Triplex for Small-Molecule Detection

Regarding small molecules, Ma et al. devised a **ThT** “switch-on” assay, where bleomycin cleaves a preformed hairpin molecular beacon to release G3 DNA and induce **ThT** fluorescence, to detect the chemotherapeutic agent bleomycin [52]. In this assay, bleomycin-Fe(II) cleaves a preformed G3 hairpin, releasing a G-rich fragment that self-assembles into a G3-**ThT** complex, yielding fluorescence. Compared to G4 DNA, G3 DNA had lower background fluorescence and higher response to bleomycin activity.

The unique interaction between G3 and **MB** has been exploited in electrochemical platforms. Zhao and co-workers discovered that **MB** stabilizes the G3 fold and decreases its diffusion current more strongly than with G4, enabling the first G3/**MB** electrochemical biosensor for cocaine detection [53]. Building on this principle, Chen et al. [124] developed a miniaturized homogeneous electrochemical platform that coupled an integrated microelectrode with a G3/**MB** hairpin system for melamine sensing. In this design, melamine binding induced release of a G3 segment, which bound **MB** and altered the electrochemical signal, allowing detection in the 100 μM–2.5 mM range with a 100 μM limit of detection [124].

Fluorescence-based aptasensors have also taken advantage of G3 DNA luminescence readouts. Huang et al. reported the first use of a G31/Tb^3+^ luminescent system, coupled with RCA, to detect the veterinary antibiotic ofloxacin, with a detection range of 5 pM to 1 nM and limit of detection of 0.18 pM [125]. They found the system to be selective against other veterinary antibiotics and capable of testing in actual food samples. Related approaches using G3-**ThT** interactions have been adapted for other small-molecule antibiotics: G31-**ThT** was used for kanamycin detection, in which the detection range for kanamycin was 50–2000 nM with a limit of 1.05 nM without the use of an amplification strategy [126]. Zhou-Jie Liu et al. developed an assay for detection of vancomycin (VCM), a glycopeptide antibiotic, utilizing the **ThT**-G3 interaction and a vancomycin binding aptamer containing putative G3 portion [127].

In an example of mycotoxin applications, the G31-**ThT** interaction was used with an aptamer for ochratoxin A detection [128]. The assay had a linear detection range of 0–0.71 ng/mL and an LOD of 0.059 ng/mL (4.7-fold lower than equivalent G4), was selective against other mycotoxins, and was capable of testing in real food samples with recovery rates of 96.3–103.5%. The same group next utilized the G31-**ThT** interaction, combined with an aptamer and hybridization chain reaction amplification strategy, to develop ochratoxin A detection [129]. This rendition had a linear range of 0.02–2 ng/mL and an LOD of 0.008 ng/mL, was selective against other mycotoxins, and could detect ochratoxin A in spiked food samples with recovery rates > 95%.

### 4.5. G-Triplex-Based Metal Ion Detection

Recently, G-triplex-based biosensors have been applied with the aim of detecting metal ions. In one instance, specific qualitative identification of chromium species was achieved using an HtelG3-based fluorescence turn-on assay [130]. Here, chromium-induced G3 unfolding was measured through the release of coralyne, a fluorophore that may be quenched upon intercalative binding to triplex DNA [131]. In another example, G3 and G4 peroxidase-mimicking DNAzymes were utilized to detect heavy metal ions [132]. Here, the diverse effect of various metal ions on G3/G4 structure formation (and consequent peroxidase activity) was exploited to establish a unique fingerprint for seven different heavy metal ions. This sensor array was employed to identify heavy metal presence in tap water in a cheap and effective manner.

Multiple studies have utilized a hairpin molecular beacon composed of a thymine-rich stem and 3′ G3 sequence to develop a label-free means of detecting Hg^2+^ ions, reduced glutathione, and glutathione reductase activity in a single label-free assay [133]. Here, hairpin formation was mediated through the association of Hg^2+^ to form stable T-Hg^2+^-T base pairs within the stem. This Hg^2+^-induced hairpin formation takes up one of the G-tracts and thus prevents G3 formation and consequent **ThT** fluorescence. Upon the addition of glutathione, an antioxidant that is regenerated by glutathione reductase and competes for Hg^2+^, G3-induced **ThT** fluorescence may be recovered. This system could detect Hg^2+^ concentrations to a limit of 20 nM and glutathione reductase activity as low as 0.01 mU/mL. Hui Zhao and colleagues detected Hg^2+^ using the same principle, with a range of 1–100 nM, to a limit of 0.2 nM [134]. Furthermore, the beacon was selective against other metal ions and used to test for mercury in cigarettes, tap water, and river water. Most recently, Cai et al. developed another Hg^2+^ biosensor using G31 and the same detection principles [87]. They achieved a linear range of 0–300 nM, a limit of detection of 5.32 nM, selectivity against other cations, and functionality in testing water and milk samples spiked with Hg^2+^.

Utilizing the specific propensity of calcium to stabilize G3 DNA, our group utilized cMYC-G3 and the chemiluminescent oxidation of luminol to devise a Ca^2+^ biosensor [54]. In this design, Ca^2+^ modulated the folding of G3 and subsequent hemin-mediated catalysis, enabling sensitive readouts of calcium levels with potential biological relevance. Finally, Xiao-Yu Li and colleagues developed a rapid, label-free biosensor for Pb^2+^ ion detection using the G31′-**ThT** interaction [135]. Here, Pb^2+^ binding enhanced G3 folding and fluorescence, delivering ultrasensitive detection with environmental relevance for monitoring lead contamination.

## 5. Conclusions and Future Directions

The study of G-triplex nucleic acids has rapidly evolved from initial interest as proposed intermediates of G-quadruplex folding to the current recognition that G-triplex nucleic acids are distinct, functional structures with biological and technological relevance. The study of G-triplex structures remains tightly linked to that of G-quadruplexes through multiple threads. Understanding of the role of G3 structures in the folding dynamics of G4, originally envisioned in a simple multi-state system involving G3 on the pathway to G4, has evolved. More recent studies have proposed a role for G3 as off-pathway intermediates, often quite transient when compared to misfolded G4 intermediates. None the less, it is clear that in some cases, truncated G4-forming sequences can give rise to stable G3 structures. However, as G-triplex-forming sequences are G-rich, the propensity to form multimeric G-quadruplex structures must always be considered. Unfortunately, this has not been uniformly addressed in the literature, leading to some questions as to the nature of the structures that are being studied. In analogy with G-quadruplexes it is likely that G-triplexes also can adopt multiple folds and loop topologies. The aspect of polymorphism is further complicated by the possibility of formation of other, unimolecular structures such as G-hairpins. Indeed, the relative thermal instability of G-triplex structures and NMR studies demonstrating a plurality of structures formed from many nominal G-triplex-forming sequences highlight the challenge of polymorphism. It is anticipated that ongoing studies will reveal a structural diversity of G3 that rivals that of G4 and will provide insights into the origin and preferences of G3 formation and polymorphism. A related challenge concerns questions about generalities and rules governing G3 formation. While it has been established that some truncations of G4-forming sequences result in stable G3 formation, many others do not and the specific sequence rules for G3 formation remain unclear. Similarly, environmental effects on G3 formation are not well-established, and even fundamental questions on the nature of cation association remain to be addressed. Without firm rules guiding the sequence motifs most likely to form stable G3 structures, bioinformatics approaches to delineate the frequency and distribution of these motifs may be somewhat premature; however, these will provide valuable data to address an important question: what are the biological roles, if any, of G3 structures? In the context of DNA, these roles may arise in contexts where G3 formation competes with duplex formation, perhaps with the contribution of i-motif formation on the complementary strand, either independently or in competition with formation of G4 structures at the G3 locus. The identification of specific interactions between proteins such as BRCA2 and G3 structures provide support for a role for these structures in processes such as G4 remodeling. Highly G3-selective ligands will play an important role in elucidating the biology of G3; however, there have been no reports yet of such compounds. In contrast, a number of well-established G4 ligands have been shown to also bind to G3 structures. To the extent that G3 does play a biological role, the lack of selectivity of these G4 ligands casts doubts on the origin of the biological responses observed for these compounds. Clearly, there is an unmet need for highly selective G3 ligands and better characterization of established G4 ligands for G3 binding.

One ligand that binds to both G4 and G3 is hemin, and this has led to many studies of peroxidase activity catalyzed by G3–hemin complexes, in analogy to those of G4–hemin. The specific advantages of the G3–hemin systems have not fully been explored, although the fact that these require smaller sequence DNAzymes and provide longer duration signals compared to their G4 counterparts is encouraging. Perhaps more significant is the report of G3 DNA catalysis of cycloaddition reactions. Here, one can imagine distinct advantages of G3-based DNAzymes compared to G4. For example, while the specifically bound cations of G4 are sequestered between G-tetrads, in the case of G3, specifically bound cations appear to be more accessible and thus may be exploited in designed metallo-DNAzymes. One caution here is that many of the reports of G3-based catalysis have not adequately addressed the possible role of multimeric G4 structures in the observed rate acceleration.

The peroxidase activity of G3–hemin and the ability to sense G3 structures via turn-on fluorescence using ligands such as **ThT** has led to a remarkable interest in G3-based biosensors. A wide range of amplification and detection modes have been reported for analytes ranging from metal ions to nucleotide sequences, to small molecules and proteins. In the few cases where these G3-based biosensors have been directly compared to the corresponding G4-based biosensors, some advantages of G3 have been identified and attributed to the increased ease with which these are released from the shorter, C-rich complement strand. However, in many cases the G3 element that is proposed to be involved in signal generation has not been well characterized and the observed signals not disambiguated from those arising from other potential structures.

It has been just over a decade from the initial report of an experimentally observed G-triplex DNA structure. In this brief period, interest in G3 DNA and RNA structures has seen tremendous growth. Still, many challenges remain, and there is much fundamental work that remains to be carried out. While G3 structures have been widely adopted in biosensing applications, the biological relevance and therapeutic potential of targeting these structures are not well established. However, with the close association of G3 with the better established target G4, and in analogy with other non-canonical structures, the chances are high that G3 will reveal itself to be a key player in biological processes and a focus of drug discovery as more is discovered about the newest member of the non-canonical nucleic acid family.

## 6. 2025 Advances in G-Triplex and Related Structures

Though this review is primarily focused on G-triplex literature through the year 2024, several papers have since been published that provide unique insights into G-triplex formation and applications. Xingtong Liu et al. utilized a polyaniline-based hybrid membrane to embed α-hemolysin, which enabled direct nanopore detection of G31′ [136]. Furthermore, this detection method could differentiate between parallel and antiparallel folds. Tao Li et al. provided in vitro evidence for G-triplex formation of TERRA (Table 2) and took the first steps in developing an rG3-based nucleic acid biosensor, demonstrating that Cas13a can effectively trans-cleave the TERRA rG3 [137]. Using CD, NMR, and molecular modeling, they demonstrated that TERRA forms a stable, parallel rG3 with three G-triad planes stabilized by K^+^. Furthermore, TERRA rG3 binds **ThT**, *N*-methyl mesoporphyrin IX, and hemin, displaying fluorescence and peroxidase-like activity. Michal Janeček and coworkers [138] presented an all-atom, enhanced-sampling simulation protocol reproducing GQ folding from single-strand to native parallel G4. Using sequences containing two, three, and four G-tracts, the pathway proceeds through a G-triplex intermediate via a multichannel pathway, corroborating triplex intermediates as kinetically stable steps within the broader kinetic-partitioning mechanism [138].

## Figures and Tables

**Table 1:** Full-length and truncated G4 DNA sequences discussed in text.

Name	Sequence	Origin
Tel22	A GGG TTA GGG TTA GGG TTA GGG	Human telomeric sequence
Tel21	TTA GGG TTA GGG TTA GGG TTA	Truncated human telomeric sequence
Tel15	GGG TTA GGG TTA GGG	Truncated human telomeric sequence
TBA	GG TT GG TGT GG TT GG	Selex
T1	GG TT GG TGT GG	3′ truncation of TBA
htel1	TA GGG TTA GGG TTA GGG TTA GGG	Human telomeric sequence
CatG4	T GGG TA GGG C GGG TT GGG AAA	Selex
G31	T GGG TA GGG C GGG	3′ truncation of CatG4
G31′	T GGG AA GGG A GGG	Alteration of G31
EAD2	CT GGG A GGG A GGG A GGG A	Designed sequence
G3-A	CT GGG A GGG A GGG A	3′ truncation of EAD2
cMYCPu27	TGG GGA GGG T GGG GA GGG T GGG GAA GG	cMYC protooncogene promoter
cMYCG3	T GGG GA GGG T GGG GAA	5′ truncation of cMYCPu27
G3-F15	TC GGG AA GGG A GGG	Modification of G3-A

**Table 2:** Full-length G4 RNA sequences discussed in text.

Name	Sequence	Origin
C	C GG C GG C GG C GG	*Arabidopsis* transcriptome
AA	GG AA GG AA GG AA GG	*Arabidopsis* transcriptome
ATR	GGG A GGG AA GGGG AA GGGG	*Arabidopsis* 5′-*UTR*
SMLX	GGGGG U GGGGGG UUA GGG UUA GGG	*Arabidopsis* 5′-*UTR*
RG1	GG CU GG CAAU GG C GG	SARS-CoV-2
TERRA	UA GGG UUA GGG UUA GGG UUA GGG U	Human telomeres

## Data Availability

No new data were created or analyzed in this study. Data sharing is not applicable.

## Abbreviations

Atomic force microscopy (AFM); 2,2-azino-bis(3-ethylbenzothiazoline-6-sulfonic acid (ABTS); G4 structure-specific antibody (BG4); bio-layer interferometry (BLI); circular dichroism (CD); Clustered Regularly Interspaced Short Palindromic Repeats (CRISPR); Dam methyltransferase (Dam MTase); differential scanning calorimetry (DSC); double-stranded specific nuclease (DSN); duplex-specific nuclease signal amplification (DSNA); density functional theory (DFT); *Escherichia coli* (*E. coli*); Epstein–Barr virus (EBV); Escherichia coli RY13 (EcoRI); enzyme-linked immunosorbent assay (ELISA); electrophoretic mobility shift assay (EMSA); electrospray ionization mass spectrometry (ESI-MS); isothermal exponential amplification (EXPAR); exonuclease III (Exo III); fragment molecular orbital (FMO); fluorescence resonance energy transfer (FRET); FRET-point accumulation in nanoscale topography (FRET-PAINT); G-triplex DNA (G3 DNA); G-quadruplex DNA (G4 DNA); green fluorescent protein (GFP); human immunodeficiency virus (HIV); human papilloma virus (HPV); isothermal calorimetry (ITC); limit of detection (LOD); methylene blue (**MB**); molecular dynamics (MD); microRNA (miRNA); metal–organic framework (MOF); microscale thermophoresis (MST); Metadynamics (MTD); 8-methylguanine (m8G); nuclear Overhauser effect (NOE); nopaline synthase (NOS); 8-oxoguanine (oxo8G); polyacrylamide gel electrophoresis (PAGE); bisquinolinium pyridine dicarboxamide (**PDC-biotin**); rolling-circle amplification (RCA); residual dipolar coupling (RDC); RNA G-triplex (rG3); RNA G-quadruplexes (rG4); RNA helicase associated with AU-rich element (RHAU); severe acute respiratory syndrome coronavirus 2 (SARS-CoV-2); benzo[c]phenanthridine alkaloid sanguinarine, cationic form (**SG+**); benzo[c]phenanthridine alkaloid sanguinarine, alkanolamine form (**SGOH**); single-molecule FRET (smFRET); selective 2′-hydroxyl acylation with Li^+^-ion primer extension (SHALiPE); single-molecule pull-down (SiMPull); surface plasmon resonance (SPR); surface using scanning tunneling microscopy (STM); thrombin binding aptamer (TBA); tetraethylene glycol (TEG); thioflavin T (**ThT**); temperature-jump (T-jump); vancomycin (VCM); 8-oxo-7,8-dihydroguanine (8-OG); 9-ethylguanine (9eG).
